# Setting the One Health Agenda and the Human-Companion Animal Bond

**DOI:** 10.3390/ijerph111111110

**Published:** 2014-10-27

**Authors:** Gregg K. Takashima, Michael J. Day

**Affiliations:** 1 GKT Enterprise, PC. Lake Oswego, OR 97035, USA; 2 School of Veterinary Sciences, University of Bristol, Langford BS40 5DU, UK; E-Mail: M.J.Day@bristol.ac.uk

**Keywords:** human-animal bond, one health, pets, animal-assisted therapy, dogs, heart disease, cancer, autism, public health

## Abstract

“One Health”, also called “One Medicine”, began as an initiative advocating greater integration of human and animal medicine, in the 1800s. This concept has recently come to prominence, driven by the recognition that 75% of the newly emerging infectious diseases will arise from animal reservoirs, and that successful control and prevention will require a coordinated human medical and veterinary approach. Consequently, many One Health discussions have centered on the surveillance of animals in order to anticipate the potential emergence of new zoonotic diseases. An area that has been given only cursory mention, are the many ways that small companion animals benefit individual, community and possibly world health. The goal of this paper is to briefly review some of the evidenced-based data concerning the benefits of having companion animals in our lives, focusing on four major areas; cancer, heart disease, autism spectrum disorder (ASD), and the potential positive economic effects of the human-companion animal bond on One Health. Heart disease and cancer are the two leading causes of morbidity and mortality in the world, while ASD is a growing concern, not only for its individual effects, but also for its effect on family units, educational institutions, and its social implications for the community. In addition, these diseases can greatly affect the national and global cost of healthcare, as well as the economic output of a nation. It is therefore important to include and build on the concept of the Human-Animal Bond (HAB) as it relates to healthcare in these areas.

## 1. Introduction

The concept of “One Health” calls for the close integration of human, animal, environmental and ecosystem health. The first inklings of such an association can be traced back to the early days of the ancients, where healers often treated both humans and animals [1]. In the 11th–13th centuries, the Chinese maintained a collaborative health program for both humans and animals [2]. Later, in 18th century France, Claude Bourgelat, considered the father of veterinary education, recommended the comparative approach to human and animal medical science [2]. In the 19th century, with the dawn of microbiology and cellular pathology, scientists such as Rudolf Virchow also advocated a comparative approach to link veterinary and human medicine [3]. After this time, both human and veterinary medicine appeared to pursue separate paths and little interdisciplinary cooperation was noted in the early 20th century. Even though the term “one medicine” had been proposed sometime earlier, it was Calvin Schwabe’s recognition in 1976, of the close association between animal and human medicine that brings us to our current status of One Health [1].

Today, if you open the website of the growing number of One Health Organizations (e.g., the One Health Commission, One Health Initiative, One Health: CDC, to name a few) you will note a major focus in One Health has been the transmission of infectious disease between wild and domestic animals including pets and humans, for example the study; *Surveillance of zoonotic infectious diseases transmitted by small companion animals, by Day M.J., et al.* [4]. However, in the case of our small companion animals, (of a wide range of species, but most typically pet dogs and cats), “One Health” means so much more than this.

Small companion animals live in close association with human families in most cultures and countries of the world. The numbers of such animals are significant, with, for example, an estimated 70 million pet dogs and 74 million pet cats in the USA alone [5]. In 2010, the World Small Animal Veterinary Association established a One Health Committee [6] to highlight the significance of small companion animals within the global One Health Agenda.

Small companion animals are of direct One Health importance due to the benefits to human health these species provide. Dogs in particular, develop a spectrum of spontaneously-arising degenerative, neoplastic, allergic and autoimmune disorders that provide unique models for the investigation of the counterpart human diseases [7]. Major advances are now being made in canine genomics with “translational research” from comparative canine medicine spilling over into developments for human healthcare [8,9]. The more immediate and long-term benefit to human wellbeing comes from the Human-Companion Animal bond. This aspect of the interaction between man and animals is now an area of active investigation and this short paper provides selected examples of such studies.

### Human and Companion Animals: A Historical Perspective

The Human-Animal Bond (HAB) has been defined as “the dynamic relationship between people and animals such that each influences the psychological and physiological state of the other” [10]. In the case of small companion animals, this relationship likely goes back to the time of domestication of the dog some 35,000 years ago. Throughout history, there are numerous examples of the important role of dogs and cats in society, religion, art and science and early recognition of the benefits of interaction with these companion animals. Formal recognition of the benefits to human health of keeping small companion animals goes back to the 18th century [8]. In the 19th century, animals were commonly found in mental health institutions and the social reformist and nurse Florence Nightingale advocated that the chronically ill should keep “a small pet” for an increased sense of well-being [11,12]. In the past 25 years there have been major advances in the formal scientific documentation of such benefits and there is much contemporary research into the Human–Companion Animal Bond.

## 2. Methodology

A journal review using PubMed, HABRI Central, Pet Partners library collection, as well as the 2013 IAHAIO (International Association of Human-Animal Interactions Organization) conference proceedings, was conducted from January 2012 through August 2014. Articles were selected based on research primarily pertaining to four major areas; that of cardiology, cancer, autism and economic and public health benefits of the HAB

These areas were chosen primarily for their significant global effect on the human population in morbidity and mortality (cancer and heart disease) and by the (in the author’s opinion) growing amount of research involving the HAB in these particular fields. Economics was included as this appears as a topic world-wide as it relates to the effects, politics and delivery of healthcare.

Key words used in these searches were: Human-Animal Bond; One Health; Pets; Animal-Assisted Therapy; Dogs; Heart Disease; Cancer; Autism; Public Health.

## 3. Four Major Research Areas

### 3.1. Heart Disease and the Human-Companion Animal Bond

Heart-related disease is the leading cause of human death in the world, accounting for approximately 17 million deaths yearly [13]. Since the 1980s when research documented an association between pet ownership and a significant decrease in mortality one year following a coronary event [14], the therapeutic value of animals has been increasingly studied. A current review of the research, while not always consistent [15,16], suggests that pet ownership and/or positive interactions with an animal can reduce or affect the risk factors (*i.e*. physical inactivity, obesity, hypertension, hyperlipidemia, stress and depression) associated with cardiovascular disease [15,16].

In a review of the research on positive interactions with animals and human cardiovascular disease, the American Heart Association published this statement in May 2013: “Pet ownership, particularly dog ownership, may be reasonable for reduction in cardiovascular disease risk (CVD)” [17]. A summary of the American Heart Association findings is given below:

- Pet ownership is correlated with lower systolic and often diastolic blood pressures. Just one of many examples, in one randomized study, ambulatory blood pressures decreased significantly (*p* < 0.001) in a patient group that adopted pet dogs. In a later follow-up study, all participants (including the “pet-deferred” group) had adopted dogs with similar reduction in systolic blood pressure [18].
- In three cited studies [19,20,21], the largest containing 5741 participants and the smallest with 32, all reported lower cholesterol and/or triglyceride levels linked to pet ownership. One study also linked non-dog ownership with increased diabetes and tobacco use [21].
- Dog ownership was associated with increased physical activity in studies performed in such culturally dissimilar regions such as Canada, Australia and Japan [22,23,24].
- Due to the increased physical activity of many dog owners, the level of obesity appears to be reduced in most studies. One mechanism whereby dog ownership may assist in weight management programs is the role they play in social support, which is a powerful predictor of adoption and maintenance of behavior change (e.g., a weight loss program). Besides providing encouragement and motivation to walk, concerns about safety while out walking may be reduced [23,25,26].
- Pet ownership may be an independent modulator of cardiac autonomic imbalances. The mechanisms responsible for this interaction are complex, but the current hypothesis links improved mood and emotional state to decreased central and regional autonomic activity, improved endothelial function and thereby more appropriate blood pressure and reduced cardiac arrhythmias, with pets conferring more significant positive effects than drugs [27,28,29].
- Cardioprotective effects may be conferred on pet-owners *versus* those without pets. Independent of the severity of cardiovascular disease, dog ownership in one study decreased the mortality of cardiovascular re-occurrence by ~ fourfold [30].

Additionally, in a canine assisted ambulation (CAA) study, relative to CVD, a decreased refusal of the patients to early ambulation was documented, with resulting shorter hospital stays and improved outcomes [31].

### 3.2. Cancer and the Human-Companion Animal Bond

Cancer is another leading cause of human death and morbidity in the world, with approximately 8.2 million deaths and 14.2 million cases diagnosed in 2012 [32]. Positive interactions with animals may be beneficial for many going through cancer therapy, with patients reporting improved perceived health and decreased depression [33,34]. Additionally, improved arterial oxygen saturation levels and positive influences on the psychosocial well-being of patients have been reported [35].

In pediatric cancer studies, it was reported that interactions with therapy animals were beneficial in a number of ways:

- Normalizing the hospital experience: children demonstrated an improved acceptance of the hospital experience, that it was “more like home”, and that they felt “less ill”, and even happier, when interacting with the animal therapy team [36,37,38,39].
- Improved motivation to participate in the treatment protocol, to maintain their motivation over time, and to want to “get better” or stay optimistic [39,40].
- Distracted or lessened worry, anxiety, unhappiness and pain, which in turn improved comfort level and provided some degree of joy [39,41].

In an investigational epidemiological study involving 1591 Non-Hodgkin Lymphoma cases and 2515 controls in the San Francisco area, pet ownership was associated with a reduced risk for Non-Hodgkin’s lymphoma and diffuse large-cell lymphoma [42]. Possible explanations may be the association between pet ownership and altered immune function and desensitization to allergens, given that immune-competence and immune-related activity and the association with Non-Hodgkin’s Lymphoma are well accepted. In addition to providing comfort, relief and potential cancer risk reduction; some animals appear to have the ability to identify people suffering from bladder, ovarian, lung, prostate and breast cancers through olfactory signals; providing a potential valuable (and less invasive) addition to human cancer diagnosis [43,44]. As in many diseases, early detection can provide for a more efficient and rapid intervention and management of this disease.

### 3.3. Autism and the Human-Companion Animal Bond

Autism spectrum disorder (ASD) is a socially debilitating neurodevelopmental disorder that is estimated to affect 1 in 88 children in the USA [45]. ASD involves impairment of social development, associated communication deficits, depressed interest and repetitive behaviors, and as such is a highly disruptive and deeply felt event for families of such individuals. Ever since the seminal presentation by Boris Levinson in 1961, where he proposed that interactions with dogs might improve social communication of ASD children, and later, the publication of his book, *Pet-Oriented Child Psychotherapy*, where he again advocated for what is now called Animal Assisted Therapy (AAT) [46], there has been gradual, and now, widespread incorporation of AAT into ASD therapy. Reported benefits include improved prosocial behaviors, self-efficacy, motivation to engage and improved emotional connections to other family members [47].

Research into the dynamics of the processes that appear to effect these changes in ASD and other conditions related to human health is ongoing. A few examples of recent research findings may illuminate how positive interactions with animals may effect these changes:

- A South African study demonstrated increases in β-endorphins, oxytocin, prolactin, β-phenylethylamine and dopamine after positive interactions with dogs [48]. These hormones have been associated with blood pressure regulation, analgesia, stress relief, joy, pleasure and bonding behavior.
- Elevated levels of oxytocin have been particularly associated with positive interactions with animals and oxytocin is seen to be a potentially key neuropeptide in ASD. Increased oxytocin levels are associated with improved bonding and interactions with others, more appropriate trusting, less repetitive behaviors, reduced aggression, more empathy and improved learning [49].

### 3.4. Economic Benefits of the Human-Companion Animal Bond

In 1997–2009, the global economic impact of zoonotic infectious diseases was estimated at US $80 billion [50]. Economic losses from diseases that could benefit from AAT or pet companionship are equally significant. In the USA alone, cardiac disease accounted for an estimated $444 billion in direct cost and lost productivity in 2010 [51]. Human health savings of $3.86 billion over 10 years have been linked to pet ownership as related to a decrease in doctor visits in studies in Australia and Germany [52], and in a separate study, savings of 175 million annually was estimated if Australian dog owners would all walk their dogs for 30 min each day [53]. The potential positive effects of pets on cardiac disease (as described above) and cancer alone could be extremely significant, but in addition, animals are reported to have positive effects on a wide spectrum of health and social issues including wound healing and immune health through the effects of the neurotransmitter oxytocin [49,54,55,56], pediatric respiratory diseases [57], child development [58], elder care [59,60,61] and pain reduction or distraction [39]. Pets are potential influencers of community health, with some evidence they can provide a “sense of community” and improve the “social capital” of a community [62]. Pets have even been implicated to improve the motivation of people to give up smoking or lose weight [19,63].

## 4. Conclusions

The payback for recognizing and nurturing this connection between animals and humans has potential implications for the community and for individual stability and health, improved economic outputs and healthcare cost savings. Already, AAT is being put into practice by organizations such as 35+ year old Pet Partners (formerly Delta Society) [64], whose 11,000 therapy animal teams visit over 1 million patients throughout the USA and 14 other countries. The positive effects that companion animals can possibly have on our lives and communities, reinforces the need for a more prominent standing of the HAB among global One Health initiatives, research and discussions.

## Figures and Tables

**Figure A1:**
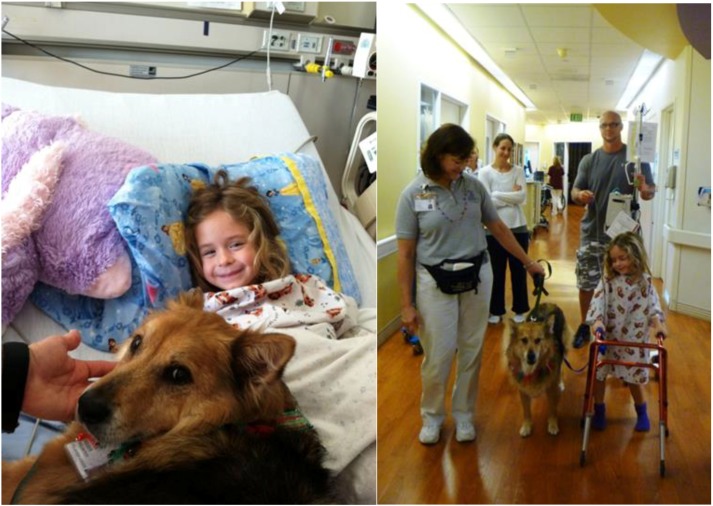
Jordan greets “Jenna” and walks with her as a first step to recovery.

## Appendix

Jordan Pollard is a little girl who has just had surgery for a rare congenital hip disease. For four days after the operation she did not want to eat or to move from her hospital bed. That was until she received a visit from “Jenna”—a trained Animal Assisted Therapy (AAT) dog. When Jordan was allowed to interact with Jenna in bed, her face lit up with a smile. Then, with the promise of a chance to “walk” Jenna around the pediatric floor, Jordan was enticed to eat the meal that she had been ignoring on her tray. This is only one of thousands of such stories of the power of the human-companion animal bond in aiding human healing, and is aptly demonstrated (Figure A1) by Jordan’s smile and her focus on Jenna while on the promised walk.

## References

[1] Schwabe C.W. (1984). Veterinary Medicine and Human Health.

[2] Driesch A., Peters J. (2003). Geschichte der Tiermedizin.

[3] Saunders L.Z. (2000). Virchow’s contributions to veterinary medicine: Celebrated then, forgotten now. Vet. Pathol..

[4] Day M.J., Breitschwerdt E., Cleaveland S., Karkare U., Khanna C., Kirpensteijn J., Kuiken T., Lappin M.R., McQuiston J., Mumford E. (2012). Surveillance of zoonotic infectious diseases transmitted by small companion animals. Emerg. Infect. Dis..

[5] American Veterinary Medical Association (2012). U.S Pet Ownership & Demographics Sourcebook.

[6] One Health Committee. http://www.wsava.org/educational/one-health-committee.

[7] Gammon K. (2012). The vetting process. Nat. Med..

[8] Lequarre A.-S., Andersson L., Andre C., Fredholm M., Hitte C., Leeb T., Lohi H., Lindblad-Toh K., Georges M. (2011). LUPA: A European initiative taking advantage of the canine genome architecture or unravelling complex disorders in both human and dogs. Vet. J..

[9] Shearin A.L., Ostrander E.A. (2010). Leading the way: Canine models of genomics and disease. Dis. Models Mech..

[10] Center for the Human Animal Bond, College of Veterinary Medicine, Purdue University. http://www.vet.purdue.edu/chab/.

[11] Palley L.S., O’Rourke P.P., Niemi S.M. (2010). Mainstreaming animal-assisted therapy. ILAR J..

[12] Nightingale F. (1969). Notes on Nursing.

[13] Kendall M. (1985). Geriatric Nursing.

[14] WHO (2013). WHO Fact Sheet N 317.

[15] Friedmann E., Katcher A.H., Lynch J.J., Thomas S.A. (1980). Animal companions and one-year survival of patients after discharge from a coronary care unit. Public Health Rep..

[16] Arhant-Sudhir K., Arhant-Sudhir R., Sudhir K. (2011). Pet ownership and cardiovascular risk reduction: Supporting evidence, conflicting data, and underlying mechanisms. Clin. Exp. Pharmacol. Physiol..

[17] Levine G.N., Allen K., Braun L.T., Christian H.E., Friedmann E., Taubert K.A., Thomas S.A., Wells D.L., Lange R.A. (2013). Pet ownership and cardiovascular risk: A scientific statement from the American Heart Association. Circulation.

[18] Allen K. Dog ownership and control of borderline hypertension: A controlled randomized trial. Proceedings of the 22nd Annual Scientific Sessions of the Society of Behavioral Medicine.

[19] Anderson W.P., Reid C.M., Jennings G.L. (1992). Pet ownership and risk factors for cardiovascular disease. Med. J. Aust..

[20] Dembicki D., Anderson J. (1996). Pet ownership may be a factor in improved health of the elderly. J. Nutr. Elder..

[21] Lentino C., Visek A.J., McDonnel K., DiPietro L. (2012). Dog walking is associated with a favorable risk profile independent of moderate to high volume of physical activity. J. Phys. Act. Health.

[22] Brown S.G., Rhodes R.E. (2006). Relationships among dog ownership and leisure-time walking in Western Canada adults. Am. J. Prev. Med..

[23] Cutt H., Giles-Corti B., Knuiman M., Timperio A., Bull F. (2008). Understanding dog owners’ increased levels of physical activity: Results from RESIDE. Am. J. Public Health.

[24] Oka K., Shibata A. (2009). Dog ownership and health-related physical activity among Japanese adults. J. Phys. Act. Health.

[25] Rhodes R.E., Murray H., Temple V.A., Tuokko H., Higgins J.W. (2012). Pilot study of a dog wlaking randomized intervention: Effects of a focus on canine exercise. Prev. Med..

[26] Cohen S. (2004). Social relationships and health. Am. Psychol..

[27] Allen K., Shykoff B.E., Izzo J.L. (2001). Pet ownership, but not ACE inhibitor therapy, blunts home blood pressure responses to mental stress. Hypertension.

[28] Allen K., Blascovich J., Mendes W. (2002). Cardiovascular reactivity and the presence of pets, friends, and spouses: The truth about cats and dogs. Psychosom. Med..

[29] Aiba N., Hotta K., Yokoyama M., Wang G., Tabata M., Kamiya K., Shimizu R., Kamekawa D., Hoshi K., Yamaoka-Tojo M. (2012). Usefulness of pet ownership as a modulator of cardiac autonomic imbalance in patients with diabetes mellitus, hypertension, and/or hyperlipidemia. Am. J. Cardiol..

[30] Friedmann E., Thomas S.A. (1995). Pet ownership, social support, and one-year survival after acute myocardial infarction in the Cardiac Arrhythmia Suppression Trial (CAST). Am. J. Cardiol..

[31] Abte S., Zucconi M., Boxer A. (2011). Impact of canine-assisted ambulation on hospitalized chronic heart failure patients’ ambulation outcomes and satisfaction: A pilot study. J. Cardiovasc. Nurs..

[32] Ferlay J., Soerjomataram I., Ervik M., Dikshit R., Eser S., Mathers C., Rebelo M., Parkin D.M., Forman D., Bray F. (2013). GLOBOCAN 2012 v1.0, Cancer Incidence and Mortality Worldwide: IARC Cancer Base No. 11 [Internet].

[33] Johnson R., Meadows R.L., Haubner J.S., Sevedge K. (2008). Animal-assisted activity among patients with cancer: Effects on mood, fatigue, self-perceived health, and sense of coherence. Oncol. Nurs. Forum.

[34] Orlandi M., Trangeled K., Mambrini A., Tagliani M., Ferrarini A., Zanetti L., Tartarini R., Pacetti P., Cantore M. (2007). Pet therapy effects on oncological day hospital patients undergoing chemotherapy treatment. Anticancer Res..

[35] Toro D., del Pilar Valdes M. (2010). Animal-assisted therapy as an approach to psychosocial symptoms in oncopediatric patients. Pediatr. Blood Cancer.

[36] Bardill N., Hutchinson S. (1997). Animal-assisted therapy with hospitalized adolescents. J. Child Adolesc. Psychiatr. Nurs..

[37] Gagnon J., Bouchard F., Landry M., Belles-Isles M., Fortier M., Fillion L. (2004). Implementing a hospital-based animal therapy program for children with cancer: A descriptive study. Can. Oncol. Nurs. J..

[38] Skeath P., Fine A.H., Berger A., Fine A.H. (2010). Increasing the effectiveness of palliative care through integrative modalities: Conceptualizing the roles of animal companions and animal-assisted interventions. Handbook on Animal-Assisted Therapy: Theoretical Foundations and Guidelines for Practice.

[39] Sobo E.J., Eng B., Kassity-Krich N. (2006). Canine Visitation (Pet) Therapy: Pilot study data on decrease in child pain perception. J. Holist. Nurs..

[40] Barker S.B., Wolen A.K. (2008). The benefits of human-companion animal interaction: A review. J. Vet. Med. Ed..

[41] Matuszak S. (2010). Animal-facilitated therapy in various patient populations: Systematic literature review. Holist. Nurs. Pract..

[42] Trahan G.J., Bracci P.M., Holly E.A. (2008). Domestic and farm-animal exposures and risk of non-hodgkin’s lymphoma in a population-based study in San Francisco bay area. Cancer Epidemiol. Biomark. Prev..

[43] Horvath G., Jäverud G., Jäverud S., Horvath I. (2008). Human ovarian carcinomas detected by specific odor. Integr. Cancer Ther..

[44] Moser E., McCulloch M. (2010). Canine detection of human cancers: A review of methods and accuracy. J. Vet. Behav..

[45] Baio J. (2012). Prevalence of autism spectrum disorders—Autism and developmental disabilities monitoring network, 14 sites, United States, 2008. MMWR.

[46] Levinson B. (1969). Pet Oriented Child Psychotherapy.

[47] Taylor R.R., Kielhofner G., Smith C., Butler S., Cahill S.M., Ciukaj M.D., Gehman M. (2009). Volitional change in children with autism: A single case design study of the impact of hippotherapy on motivation. Occup. Ther. Ment. Health.

[48] Odendaal J.S.J., Meintjes R.A. (2003). Neurophysiological correlates of affiliative behavior between humans and dogs. Vet. J..

[49] Beetz A., Uvnäs-Moberg K., Henri J., Kotrschal K. (2012). Psychosocial and psychophysiological effects of human-animal interactions: The possible role of oxytocin. Front. Psychol..

[50] The World Bank (2012). People, Pathogens, and our Planet. Volume 2. The Economics of One Health.

[51] American Heart Association Heart Disease and Stroke Prevention Addressing the Nation’s Leading Killers. At a glance, 2011. http://www.cdc.gov/chronicdisease/resources/publications/aag/pdf/2011/heart-disease-and-stroke-aag-2011.pdf.

[52] Heady B., Grabka M., Kelley J., Reddy P., Tseng Y.-P. (2002). Pet ownership is good for your health and saves public expenditure too. Australian and German longitudinal evidence. Aust. Soc. Monit..

[53] Bauman A.E., Russell S.J., Furber S.E., Dobson A.J. (2001). The epidemiology of dog walking: An unmet need for human and canine health. Med. J. Aust..

[54] Detillion C.E., Craft T.K., Glasper E.R., Prendergast B.J., DeVries A.C. (2004). Social facilitation of wound healing. Psychoneuroendocrinology.

[55] Gouin J.P., Carter C.S., Pournajafi-Nazarloo H., Glaser R., Malarkey W.B., Loving T.J., Stowell J., Kiecolt-Glaser J.K. (2010). Marital behavior, oxytocin, vasopressin, and wound healing. Psychoneuroendocrinology.

[56] Clodi M., Vila G., Geyeregger R., Riedl M., Stulnig T.M., Struck J., Luger T.A., Luger A. (2008). Oxytocin alleviates the neuroendocrine and cytokine response to bacterial endotoxin in healthy men. Am. J. Physiol. Endocrinol. Metab..

[57] Bergroth E., Remes S., Pekkanen J., Kauppila T., Buchele G., Keski-Nisula L. (2012). Respiratory tract illnesses during the first year of life: Effect of dog and cat contacts. Pediatrics.

[58] Lookabaugh S. (1998). Pets as transitional objects: Their role in children’s emotional development. Psychol. Rep..

[59] Kanamori M., Suzuki M., Yamamoto K., Kanda M., Matsui Y., Kojima E., Fukawa H., Sugita T., Oshiro H. (2001). A day care program and evaluation of animal-assited therapy (AAT) for the elderly with senile dementia. Am. J. Alzheimer’s Dis. Dement..

[60] Knight S., Edwards V. (2008). In the company of wolves: The physical, social, and psychological benefits of dog ownership. J. Aging Health.

[61] Banks M., Banks W. (2002). The effects of animal-assisted therapy on loneliness in an elderly population in long-term care facilities. J. Gerontol. A Biol. Sci. Med. Sci..

[62] Wood L., Giles-Corti B., Bulsara M. (2005). The pet connection: Pets as a conduit for social capital?. Soc. Sci. Med..

[63] Milberger S.M., Davis R.M., Holm A.L. (2009). Pet owners’ attitudes and behaviors related to smoking and second-hand smoke: A pilot study. Tob. Control.

[64] Pet Partners. http://www.petpartners.org/.

